# A non-invasive multimodal foetal ECG–Doppler dataset for antenatal cardiology research

**DOI:** 10.1038/s41597-021-00811-3

**Published:** 2021-01-26

**Authors:** Eleonora Sulas, Monica Urru, Roberto Tumbarello, Luigi Raffo, Reza Sameni, Danilo Pani

**Affiliations:** 1 University of Cagliari, Department of Electrical and Electronic Engineering, Cagliari, 09123 Italy; 2 Brotzu Hospital, Pediatric Cardiology and Congenital Heart Disease Unit, Cagliari, 09134 Italy; 3 Department of Biomedical Informatics, Emory University School of Medicine, Atlanta, GA 30322 US

**Keywords:** Echocardiography, Biomedical engineering, Congenital heart defects

## Abstract

Non-invasive foetal electrocardiography (fECG) continues to be an open topic for research. The development of standard algorithms for the extraction of the fECG from the maternal electrophysiological interference is limited by the lack of publicly available reference datasets that could be used to benchmark different algorithms while providing a ground truth for foetal heart activity when an invasive scalp lead is unavailable. In this work, we present the Non-Invasive Multimodal Foetal ECG-Doppler Dataset for Antenatal Cardiology Research (NInFEA), the first open-access multimodal early-pregnancy dataset in the field that features simultaneous non-invasive electrophysiological recordings and foetal pulsed-wave Doppler (PWD). The dataset is mainly conceived for researchers working on fECG signal processing algorithms. The dataset includes 60 entries from 39 pregnant women, between the 21^st^ and 27^th^ week of gestation. Each dataset entry comprises 27 electrophysiological channels (2048 Hz, 22 bits), a maternal respiration signal, synchronised foetal trans-abdominal PWD and clinical annotations provided by expert clinicians during signal acquisition. MATLAB snippets for data processing are also provided.

## Abstract (summary)

**Table:** 

Measurement(s)	electrocardiogram (maternal) • abdominal electrocardiogram (fetal) • Respiratory Rate • pulsed-wave Doppler (fetal heart) • heart function measurement • heart electrical impulse conduction trait
Technology Type(s)	electrocardiography • piezoresistive belt • pulsed doppler echocardiography
Factor Type(s)	gestational age
Sample Characteristic - Organism	Homo sapiens

Machine-accessible metadata file describing the reported data: 10.6084/m9.figshare.13283492

## Background & Summary

The clinical assessment of the foetal heart activity is an important step for diagnosis^1,2^, and monitoring purposes^3,4^. Different instrumental techniques can be adopted depending on the gestational age and goal of the examination, including ultrasonography (cardiotocography and echocardiography, mainly in the B-mode and M-mode^5^ and Doppler^6–9^), phonocardiography^10,11^, magnetocardiography^12–14^, invasive electrocardiography (ECG)^15^, and non-invasive foetal ECG (fECG)^16–18^.

According to the American Heart Association, although non-invasive fECG has been available for decades, its clinical introduction has been delayed for several reasons^8^, including the complex setup, a low signal-to-noise ratio (SNR) and the absence of public datasets enabling comparative evaluation of different techniques for the extraction of the fECG signal from non-invasive recordings, and featuring multiple simultaneous modalities for researchers.

Although some public datasets do exist, they have limitations for researchers working on signal processing techniques for fECG extraction^19^. For example, the DaISy dataset consists of a single 8-lead signal, 10 s long^20^. A larger number of signals can be found in the Non-Invasive Foetal Electrocardiogram Database, which includes 55 variable-duration recordings of the same participant, between the 21st and 40th week of gestation^21^. However, only three to four abdominal leads with variable placement are available, which prove inadequate for testing independent component analysis or source localisation algorithms commonly used for fECG extraction and analysis. Similar reservations hold for the Abdominal and Direct Foetal Electrocardiogram Database (ADFECGDB), which contains 4-lead (homogeneous placement) recordings from five women in labour, between the 38th and 41st weeks of gestation^22^. The advantage of this dataset is the presence of a direct (invasive) reference fECG, which can be used as a gold standard, along with the length of the traces, which are five minutes long. The most recent dataset made publicly available was proposed by Matonia *et al*.^23^, which shares signals with ADFECGDB. This dataset was acquired with the Komporel system, partly in late pregnancy (32nd to 42nd weeks) and partly during labour (between the 38th and 42nd weeks). The signals are between 5 to 20 minutes in length; however, the number of channels is limited to four abdominal leads along with scalp lead (for the labour dataset only). The raw data provided is for the direct fECG only, whereas the abdominal leads are pre-filtered and the extracted fECG is also made available. Reference annotation for foetal heartbeats is given by the direct lead for the labour dataset, and by a clinical annotation based on the output of a signal processing algorithm for fECG extraction for the pregnancy dataset. PhysioNet/Computing in Cardiology Challenge 2013 dataset also comprises 4-lead recordings (unknown placement, one minute long), acquired with different systems and including synthetic signals^24^. The main limitations of this dataset are the heterogeneity, the absence of a reference and a low number of channels. A more recent public dataset, the Non-Invasive Fetal ECG Arrhythmia Database (NIFEA DB)^25^, includes 12 recordings from foetal arrhythmic cases and 14 normal rhythm cases, where four to five abdominal channels and one chest maternal channel are available per record. Although this dataset collects irregular foetal cardiac rhythm signals, it lacks a reference signal that could be used for verification.

The characteristics of the above-mentioned datasets are listed in Table 1.

**Table 1:** Comparison of public datasets for foetal ECG research.

Dataset	Description	# channels	Duration	Details
DaIsy^20^	single pregnant woman	8 ch (5 abdominal, 3 thoracic)	10 s	Fs: 250 Hz
NIFECG-DB^21^	single pregnant woman, 55 multi-channel abdominal recordings between 21 and 40 weeks	5–6 ch (2 thoracic, 3 or 4 abdominal)	Variable duration	Bandwidth: 0.01–100 Hz; Fs: 1 kHz; 16 bits
CinC Challenge 2013^24^	75 recordings in set A, 100 recordings in set B	4 abdominal ch	1 minute	Fs: 1000 Hz, Different instruments, frequency response, resolution, and configuration.
FECGSYN^93^	1750 synthetic signals: 10 (virtual pregnant women) × 7 (conditions) × 5 (SNR levels) × 5 (repetitions)	32 abdominal and 2 maternal reference ECG	5 minutes	Fs: 250 Hz; 16 bits
ADFECG-DB^21,94^	5 participants in labour, 38 to 41 weeks of gestation.	4 electrodes around the navel, reference electrode on the left leg, 1 foetal scalp signal	5 minutes	Bandwidth: 1–150 Hz; Fs: 1 kHz; 16 bits; Digital filtering for power-line interference (50 Hz) and baseline drift removal
OB1DB^21^	>100 signals, invasive foetal ECG, uterine contractions + maternal/newborn clinical data	1 continuous invasive foetal ECG and 1 simultaneously recorded uterine muscular activity signal	several hours	
NIFEA DB^21,25^	foetal arrhythmias recordings (n = 12) and normal rhythm recordings (n = 14)	4–5 raw abdominal channels + 1 maternal chest	Variable duration	Fs: 500 Hz or 1 kHz.
.Matonia *et al*^23,95^	pregnancy (B1, n = 10) and labour (B2, n = 12) recordings	B1: 4 abdominal, B2: 4 abdominal + 1 foetal scalp	B1: 20 minutes, B2: 5 minutes	Fs: 500 Hz; 16 bits; Komporel acquisition system.
This work (NInFEA)^35^	70 traces from 40 pregnant women	24 abdominal unipolar ch + 3 bipolar thoracic ch + maternal respiration + foetal PWD traces	>6 s	Bandwidth: 0–550 Hz; Fs: 2048 Hz; 22 bits (resolution: 71.5 nV)

Overall, a standard fECG dataset should have an adequate number of leads (preferably sixteen or more^26^), homogeneous placement, appropriate signal quality and quantisation level (sixteen or more *effective number of bits*), high sampling frequency (1 kHz or higher), and alternative simultaneous modalities for cross-validation and benchmarking^26^ for algorithm development and electrophysiological research. Notably, these requirements mainly apply to datasets used for the aforementioned research needs, and not necessarily to the recording of non-invasive fECG for clinical use. In fact, some of the existing FDA/CE approved commercial devices make use of a reduced number of leads or sampling frequencies.

**Fig. 1:**
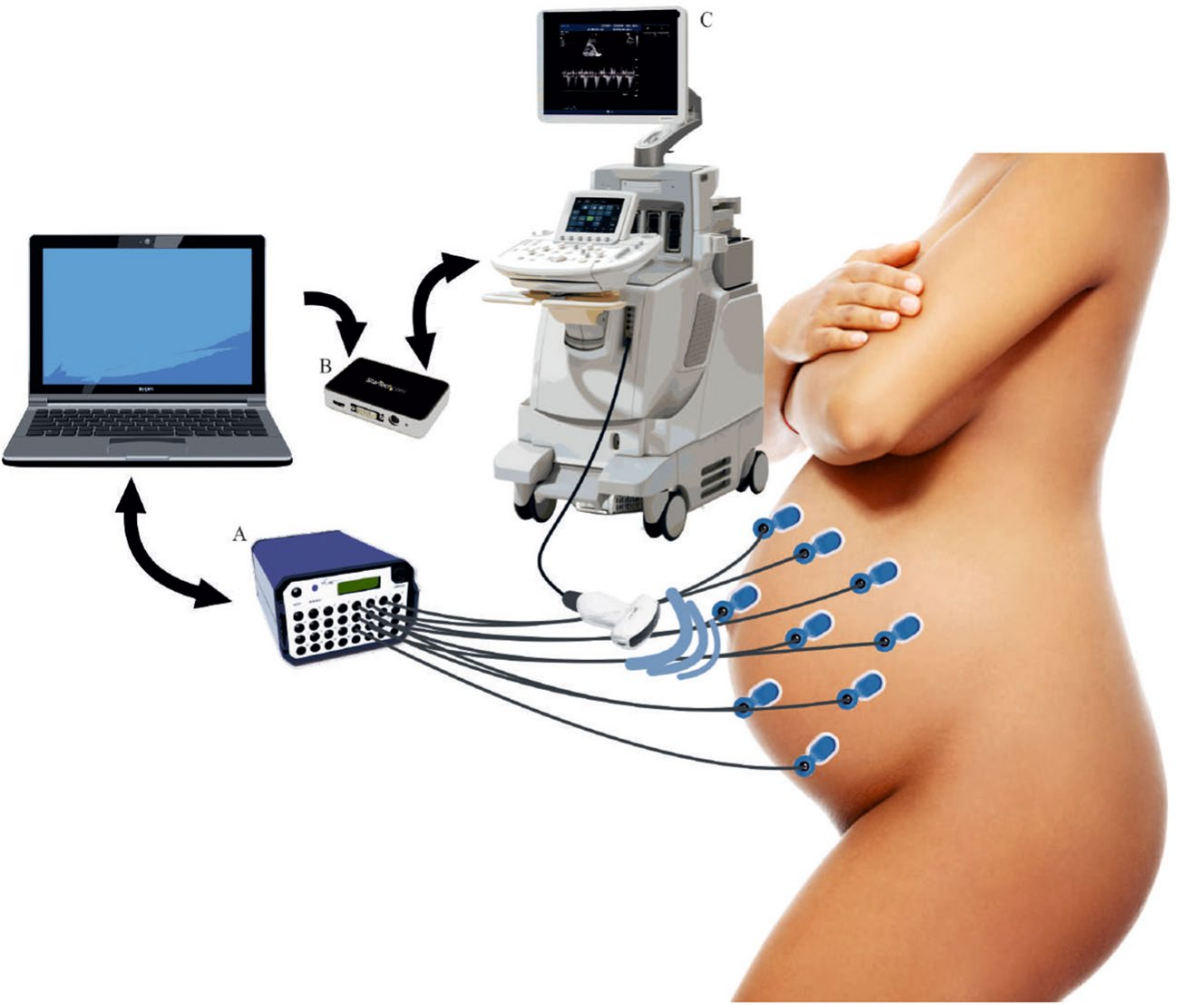
Recording setup. (**a**) Porti7 portable physiological measurement system (TMSi, The Netherlands); (**b**) USB3HDCAP USB3.0 Video Capture Device (StarTech, Ontario, Canada); (**c**) Philips iE33 Ultrasound Machine (Philips, The Netherlands).

In this study, Non-Invasive Multimodal Foetal ECG-Doppler Dataset for Antenatal Cardiology Research (NInFEA) is introduced to address the shortcomings of currently available datasets and satisfy the aforementioned features, specifically for research purposes. The dataset includes synchronised electrophysiological recordings (24 abdominal unipolar, 3 thoracic bipolar, 2048 Hz, 22 bits), maternal respiration (thoracic belt, 2048 Hz, 22 bits) and foetal cardiac PWD for the first time. A representation of the adopted setup is presented in Fig. 1. The dataset includes 60 entries from 39 pregnant women volunteers, between the 21st and 27st week of gestation. The average signal length was 30.6 s ± 20.6 s depending on the stability of the sample volume for the Doppler recording, which was inevitably affected by foetal movements. Remarkably, the sample volume stability is mandatory to achieve clinically informative representations of the atrioventricular activity. The dataset consists of a total of approximately 4000 foetal heartbeats. As the dataset was not conceived for foetal heart rate (fHR) variability studies (typically requiring at least five minutes of continuous recording), some of the records are relatively short and not recommended for signal processing algorithms requiring a long training period. Some of the cases for this database were previously used for studies on PWD^27–29^ and fECG^30–33^.

Along with the dataset, a set of MATLAB functions for data access, visualization and basic processing are also provided. Moreover, all the fECG extraction algorithms used to assess the dataset characteristics reported in this work are freely accessible on the public Git repository of the *open-source electrophysiological toolbox (OSET)*^34^, thus enabling complete reproducibility of the presented results and benchmarking with other processing approaches. The NInFEA dataset was acquired at the Pediatric Cardiology and Congenital Heart Disease Unit of the San Michele Hospital (Brotzu) in Cagliari, Italy, from healthy foetuses. The dataset is freely available on Physionet^35^.

## Methods

In this section, the recording protocol and different aspects of the dataset are described. We will also explain the supporting functions provided along with the dataset, with some informative metrics obtained for signal quality assessment of the dataset.

### Recording protocol

The dataset creation was approved by the Independent Ethics Committee of the Cagliari University Hospital (AOU Cagliari) and performed following the principles outlined in the 1975 Helsinki Declaration, as revised in 2000. All volunteers provided their signed informed consent to the protocol. During the collection of the dataset, all of the information relevant to the pregnancy was recorded. Specifically, the maternal weight and height, number of previous pregnancies, associated risk factors, gestational diabetic conditions and their prescribed treatment.

The inclusion criteria for selecting the participants were: healthy foetuses (from the cardiological perspective) and a gestational week between the 21st and the 27th. The former criterion was set to guarantee a dataset without pathological anomalies that would be poorly represented due to a reduced sample size. The latter criterion was set to obtain signals with acceptable quality in all modalities (electrophysiological and ultrasound). From a bio-electromagnetism perspective, the simple *volume conductor* approximation assumed the maternal body organs, layers of tissue and fat and amniotic fluid only attenuating the propagation of the fECG signal towards the maternal abdominal electrodes, which holds for early pregnancy but not throughout the whole gestation^36,37^. The layer with the lowest conductivity is the *vernix caseosa*, a protective waxy substance that covers the foetus that can greatly attenuate or even suppress the fECG^38^. Pioneering studies revealed how this substance presents an electrical impedance that is two orders of magnitude higher than that of amniotic fluid and foetal/maternal tissue^39^. Scientific literature in the field of electrophysiological recordings from the foetal heart explains that the vernix formation and growth generally creates a kind of blackout in the collection of the fECG signals between the 28th and (commonly) 32nd week of gestation when it begins to disappear from parts of the foetal body, in particular the face and head that come in tight contact with the maternal pelvis^40^. During this period, it is occasionally possible to record the fECG non-invasively; however, there is a significant drop in the detection rate and signal quality^41^. Moreover, the conduction paths, which were uniform before the 28^*th*^ week of gestation, become non-uniform after the 32nd week, resulting in potential morphological variations of the fECG^36^. This is due to the fact that the homogeneous volume conductor hypothesis is no longer valid, which is demonstrated by several studies including comparative analyses with foetal magnetocardiography^40^.

Since the dataset provides a mechanical reference for the foetal heart function, the highest accuracy in the fECG morphology was pursued by limiting the recordings to the early pregnancy, since in that period the problems related to vernix caseosa can be neglected. Based on these assumptions, the signals cannot be used in physiology studies to infer properties associated with other weeks of gestation. Nevertheless, the signal processing algorithms aimed at the development of fECG extraction algorithms from non-invasive recordings are usually agnostic with respect to this aspect.

Before each recording session, the cardiologist performed the medical examination as per the current clinical guidelines and screened the morphology and functionality of the foetal heart by foetal echocardiography, using the 2D B-mode, M-mode and Doppler. After the assessment of a healthy foetus and explanation of the procedures involved in the data acquisition, the informed consent was signed by the pregnant women and the acquisition setup was arranged.

Recordings were performed with the participant at rest, in a comfortable semi-sitting position on an echocardiography table. After the electrodes were attached, the respiration belt was fastened around the participant’s chest and the cardiologist checked the position of the foetus by B-mode echography (this information was included in the metadata of the dataset). The simultaneous multimodal acquisition was then initiated after the correct sample volume for the echocardiography was identified.

Among the different technologies commonly used, the foetal echocardiography is primarily used to provide a reference signal in early pregnancy. The echocardiography provides the operator with different modalities, aimed at studying different aspects of the foetal heart, including conventional 2D imaging (B-mode), pulsed-wave Doppler^42^ (or the continuous-wave Doppler, when the sample speed exceeds its limits^7^) and pulsed tissue Doppler echocardiography^43,44^, which accurately provide the localisation of many arrhythmias^45^. Additionally, the M-Mode^6,46^ along with PWD, are tipically used for the routine evaluation of fHR and rhythm, from which electrophysiological events can be roughly estimated^5^. For this dataset collection, PWD was chosen as the best option from a cardiological perspective because of its ability to accurately measure atrial and ventricular intervals. In fact, a main limitation of M-mode is that mechanical events are not well-defined and it is not possible to achieve precise measurements of time intervals between the mechanical events, making the Doppler echocardiography a superior modality for the assessment of the foetal cardiac rhythm and intervals^6^.

Cardiotocography^47^, which is typically adopted in late pregnancy for continuous HR monitoring, was discarded from the beginning. In fact, this technique is not able to provide signals useful for the detailed analysis of atrial and ventricular activity, i.e. the ultrasound signal is used to compute fHR but without morphological information on the single heart cycle, whereas foetal echocardiography is normally used for detailed analysis of the foetal heart morphology and function^48^.

The details of the PWD, respiration and foetal ECG signal acquisition protocols and the developed algorithms for pre-processing and synchronization of these different modalities are detailed in the following sections.

### Pulsed-wave Doppler

The spectral Doppler shows the blood-flow velocities, the direction of the flow, the timing of the cardiac events and the intensity of the flow. The PWD can also provide blood-flow information from a particular location in the heart or the great vessels. PWD represents the best choice to provide a reference signal for cardiac rhythm analysis^5^ in early pregnancy; as a result, it is commonly used by paediatric cardiologists for *in utero* assessment of foetal arrhythmias^7,42,45^. Of course, its accuracy in the determination of the fHR is severely limited by the temporal resolution, characteristic noises and limited definition of the 2D signal. Nevertheless, it provides adequate information on the instantaneous HR to the cardiologist and sufficient information about the atrioventricular activity and aortic flow.

#### PWD signal acquisition

**Fig. 2:**
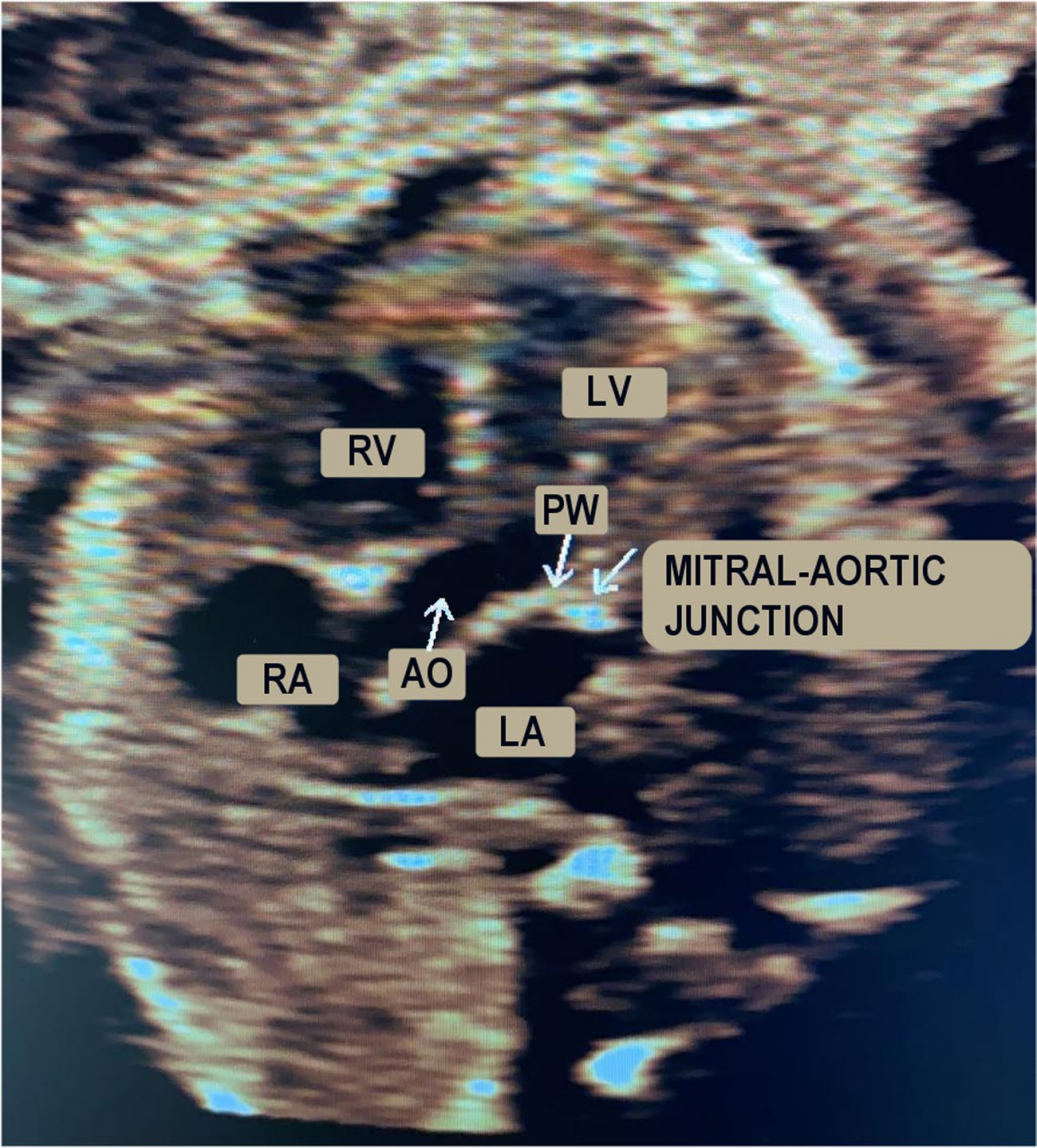
Apical 5-chamber view of the foetal heart: right ventricle (RV), right atrium (RA), left ventricle (LV), left atrium (LA), aortic region (AO), position of the sample volume for PWD (PW).

For the recording of the PWD signal, the five-chamber apical window was adopted^49^ (see Fig. 2), which allows flow monitoring across the mitral and aortic valves. In particular, an apical five-chamber view allows for recognizing the four cardiac chambers (VDX, ADX, VSN and ASN, in Fig. 2) and the first part of the aorta (AO, in Fig. 2). This window is characterised by a specific PWD pattern, typical of atrial and ventricular functions, which can be easily explained with respect to the blood flow. The atrial function gives rise to a biphasic wave, with a first peak (E) determined by the passive filling of the ventricle because of the differential pressure between the two chambers, and a second peak (A) during the atrial contraction. The E and A peaks contribute to the formation of an M-shaped waveform^50^. The systolic flow produces an opposite-polarity monophasic wave in the Doppler velocity flow (the V-wave), resulting in blood flow through the aortic valve. Note that, depending on the foetal presentation in the womb, the mitral blood inflow can direct towards the ultrasound transducer or moving away from it. Therefore, the ${\rm{E}}/{\rm{A}}-{\rm{V}}$ can have a positive polarity (positive E/A-wave, negative V-wave) or a negative polarity (negative E/A-wave, positive V-wave).

Different parameters, which are significant for the identification of heart defects and dysfunction, can be extracted from the PWD waveform^51^. Each atrial event is followed by a ventricular event in a well-defined time-interval. Normal foetuses have a 1:1 atrioventricular (AV) conduction^6^. The first sign of a conduction dysfunction is reflected in an AV block as the mechanical AV conduction time-interval could identify foetuses within this pathology^52^. Regarding the atrial activity, the E-wave is smaller than the A peak, and the ${\rm{E}}/{\rm{A}}$ ratio increases during pregnancy towards one and increases more after birth.

From a technical viewpoint, the Philips iE33 Ultrasound Machine (Philips Healthcare, The Netherlands) was used to perform the PWD measurement. The native resolution of the video was 1680 × 1050 pixels, at a frame-rate of 60 Hz with the sweep speed set to 75 mm/s. The PWD signal was recorded through the DVI output with a USB3HDCAP USB3.0 Video Capture Device (StarTech, UK). This frame-grabber was able to record 1080p HD videos with a frame-rate of up to 60 frames per second and H.264 encoding.

#### PWD processing

The whole video was converted into a single wide image using a MATLAB custom tool by (i) decomposition of the video into single frames, (ii) cropping the images to isolate the region of interest that contains only the PWD signal, (iii) identification of the useful non-redundant frames as where the update front of the PWD wraps to the leftmost side of the image and (iv) appending these frames to create a single wide image containing all of the Doppler velocity spectrum of the processed video.

**Fig. 3:**
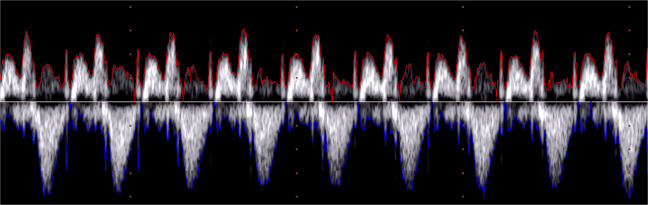
A foetal PWD, belonging to the 27^*th*^ trace, and the upper and lower envelopes automatically traced with the discussed methods. (Because of the image contrast, the reader is suggested to check the electronic version of this picture).

The single wide images representing the PWD signals are also provided with this dataset, from which various parameters, including the PWD signal envelope, can be extracted. The envelope extraction produces two signals: the upper envelope and lower envelope, respectively the red and blue lines in Fig. 3, for a part of the 27 ${}^{th}$ trace.

The envelope extraction involved the following steps, as detailed in^28^:

1. *Image binarization*: based on 2D Otsu’s method, we adopted a global threshold from a grey-level-median histogram^53^. After a test phase, this threshold was selected as the most efficient and robust regarding noise and information preservation^28^.
2. *Area opening*: removal of *four-connected* components with a small area (70 pixels). In addition to the noise, this step removed also the dotted line characterising the vertical grid of the iE33 video.
3. *Edge detection*: extraction of the two 1D envelopes, representing the upper and lower profile of the PWD image, respectively:

1 $${G}_{u}(x)=\left(\arg \mathop{max}\limits_{y}\left\{I(x,y)=1\right\}\right)-{y}_{b}$$

2 $${G}_{l}(x)=\left(arg\mathop{min}\limits_{y}\left\{I(x,y)=1\right\}\right)-{y}_{b}$$

where *y*_*b*_ is the baseline position, that is the horizontal axis line that separates the image into two parts containing the negative and the positive waves, whose polarities depend on the direction of the blood flow, if it is moving away or going towards the transducer, respectively. Figure 3 shows a segment of a the PWD signal with the associated *G*_*u*_ and *G*_*l*_ curves. Based on the time information and image resolution, the envelopes present 284 samples per second, which can be assumed to be the sampling rate for these signals.

### Foetal ECG and maternal respiration

The fECG can be ten to twenty times smaller than the maternal ECG in amplitude, and wider in bandwidth, ranging between 0.05 Hz to 250 Hz (because of QRS duration shortening caused by the higher HR)^54^. For this reason, previous research proposed minimum sampling frequencies between 1 kHz to 2 kHz and an analogue-to-digital quantization resolution of 16 bits^26^. For this dataset, the bio-potentials were recorded with a Porti7 portable physiological measurement system (TMSi, The Netherlands). The system features simultaneous sampling up to 2048 Hz on the available input channels; however, the input bandwidth is limited by the internal digital decimation filter to approximately 550 Hz (0.27 × the sampling frequency). The analogue front-end is dc-coupled (contains no high-pass filter) with a 300 mV peak-to-peak amplitude range. Therefore, the digitisation at 22 bits provides a 71.5 nV amplitude resolution. Active cables shielding and high input impedance, equal to $1{0}^{12}\Omega$, reduces the needs of hard skin preparation and power-line interference. The device features 32 simultaneously sampled channels: 24 unipolar (all of the unipolar channels are acquired with respect to their average because it implements an average reference amplifier on these channels only), four bipolar (for differential recordings) and four auxiliary (also bipolar, with an extended dynamic range, used for sensors requiring polarisation). Different versions of the adopted device were previously used for the same purpose by other researchers^55^ and more recently for advanced studies on foetal scalp electrode recordings and optimisation^56^.

**Fig. 4:**
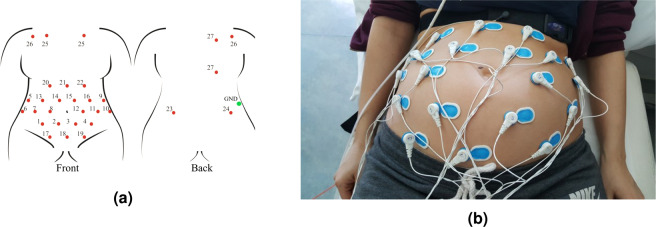
The chosen electrodes positioning for the NInFEA dataset: (**a**) front and back positioning scheme and (**b**) image of a real recording (only the abdominal channel and respiration belt are visible).

To date, there is no standard or widely accepted consensus for non-invasive fECG electrode placement. A recent review^57^, studied twenty abdominal electrode configurations, four of which used 32 or more electrodes, and the rest used less than 16. For this study, to ensure maximum versatility of the dataset and in accordance with the most popular sensor placement schemes^57^, a set of 30 signal electrodes plus a ground reference was placed as shown in Fig. 4.

**Fig. 5:**
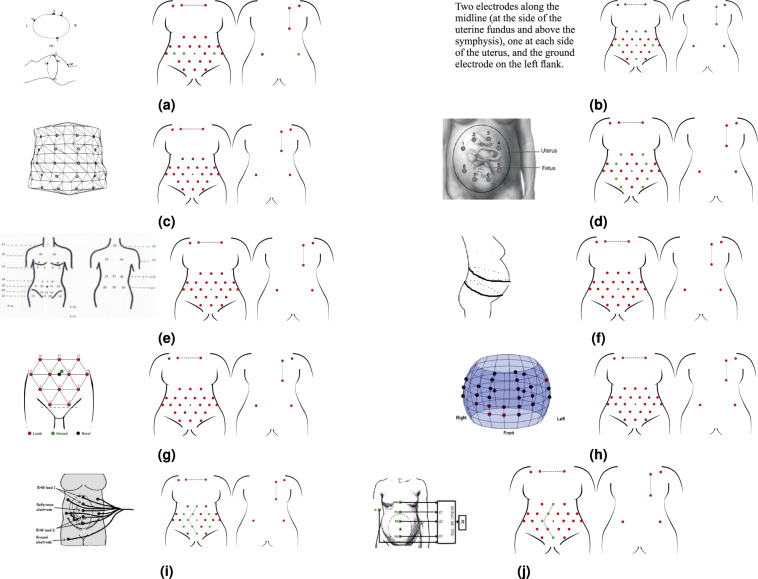
Replicability of state-of-the-art electrode positioning with the proposed dataset. In order: (**a**) Adapted from^77^, (**b**) Adapted from^78^, (**c**) Adapted from^79^, (**d**) Adapted from^80^, (**e**) Adapted from^81^, (**f**) Adapted from^60^, (**g**) Adapted from^41^, (**h**) Adapted from^18^,(**i**) Adapted from^82^, (**j**) Adapted from^83^. (The reader is suggested to check the electronic version of this picture since colours were used to identify the used (green) and unused (red) electrodes from the proposed setup to replicate the one taken from the literature.) Reproduced with permission.

**Fig. 6:**
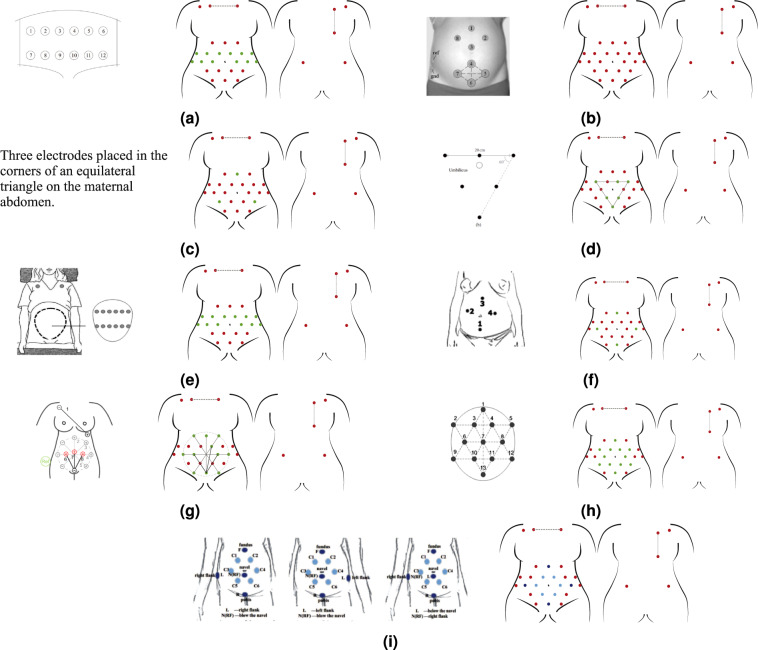
Replicability of state-of-the-art electrode positioning with the proposed dataset. In order: (**a**) Adapted from^84^, (**b**) Adapted from^85^, (**c**) Adapted from^86^, (**d**) Adapted from^87^, (**e**) Adapted from^88^, (**f**) Adapted from^89^, (**g**) Adapted from^90^, (**h**) Adapted from^55^, (**i**) Adapted from^91,92^. (The reader is suggested to check the electronic version of this picture since colours were used to identify the used (green) and unused (red) electrodes from the proposed setup to replicate the one taken from the literature.) Reproduced with permission.

This configuration can be mapped to lower-dimensional schemes by spatial sub-sampling because of its high spatial redundancy, as shown in Figs. 5 and 6. Moreover, a redundant number of electrodes also improves the chance of recording good-quality signals from multiple channels, even when the ultrasound probe placement introduces artefacts in the electrodes in its proximity. The chosen electrode configuration includes:

- 22 electrodes connected to unipolar channels, covering a large area of the maternal abdomen, avoiding iliac crests and rib regions (electrodes number 1–22, Fig. 4),
- two further electrodes connected to unipolar channels, placed on the maternal back (electrode number 23–24, Fig. 4),
- six electrodes for three bipolar channels, positioned on the maternal thorax, for recording the maternal ECG (to capture three non-coplanar maternal ECG signals, as required by some fECG extraction algorithms and adaptive filtering schemes^58–61^), electrode number 25–27, Fig. 4),
- one reference (signal ground) electrode, positioned on the right maternal hip.

**Fig. 7:**
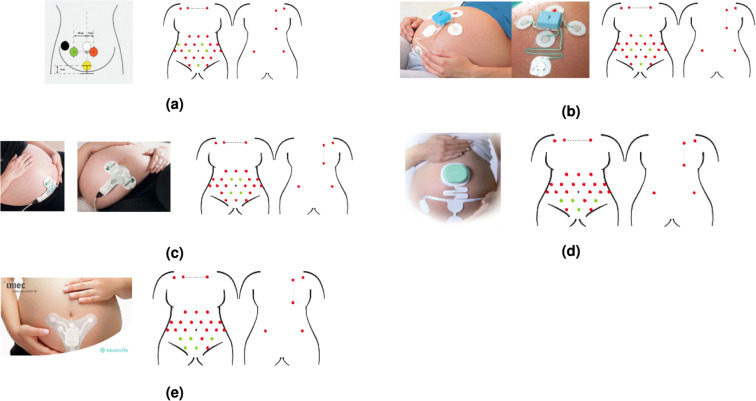
Replicability of electrode positioning used in the market-available wearable devices with the proposed dataset. In order: (**a**) Adapted from Monica AN24 (Monica Healthcare, Nottingham, UK), (**b**)Monica Novii Wireless Patch System (Monica Healthcare, Nottingham, UK), (**c**) PUREtrace (Nemo Healthcare, Veldhoven, The Netherlands), (**d**) Nemo Fetal Monitoring System (Nemo Healthcare, Veldhoven, The Netherlands), (**e**) Wearable 5-Channel ECG Chip to Monitor Fetal Heart Rate and Mobility by Imec and Bloomlife. (The reader is suggested to check the electronic version of this picture since colours were used to identify the used (green) and unused (red) electrodes from the proposed setup to replicate the one taken from the literature).

Considering the FDA/CE cleared non-invasive fECG devices present on the market, the number of channels collected in this dataset is significantly greater. In fact, except for the Meridian M110 Fetal Monitoring System (MindChild Medical, North Andover, MA, USA), which uses patches with 27 electrodes and the ground, other commercial devices use patch systems with less than six electrodes: Monica AN24 (Monica Healthcare, Nottingham, UK), Monica Novii Wireless Patch System (Monica Healthcare, Nottingham, UK), PUREtrace and Nemo Fetal Monitoring System (Nemo Healthcare, Veldhoven, The Netherlands) and the Wearable 5-Channel ECG Chip to Monitor Fetal Heart Rate and Mobility (Imec, Leuven, Belgium, and BloomLife, San Francisco, CA | Genk, Belgium). Figure 7 shows how the electrode positioning from the devices available on the market (characterised by a number of electrodes compatible with the dataset) are nearly reproducible with our setup. Remarkably, the highest number of electrodes of the dataset is meant to provide unavailable features to the scientists working on fECG extraction and foetal cardiac physiology. For instance, it can be useful for studies on the optimisation of electrode placement, the recovery of hidden information on the cardiac axis, the assessment of algorithms for the solution of the inverse problem and lead reconstruction by geometric transforms.

Small electrodes were chosen considering the large number of abdominal channels. BlueSensor N electrodes (Ambu, Denmark) have been developed for neonates, have a highly conductive liquid gel that reduces the skin-electrode contact impedance. Moreover, the offset connector helps to reduce the cable movement artefacts. Prior to electrode attachment, a mild skin treatment was performed on the maternal abdomen using NuPrep (Weaver and Company, USA), an abrasive gel enhancing the conductivity by reducing the skin contact impedance.

A piezo-resistive respiration belt was placed around the maternal chest and was connected to one of the auxiliary inputs of Porti7. The utilisation of the respiration belt enables further physiological studies and improves signal processing on the electrophysiological signals, e.g. removal of the baseline drift caused by thoracic wall movements during inspiration and expiration.

### Multimodal synchronisation

The Porti7 device and iE33 ultrasound machine cannot be directly synchronised for the acquisition of long traces. For this reason, offline synchronisation was performed on the signals recorded from the Porti7 and frame-grabber by exploiting an external trigger signal. This trigger was a low-voltage monophasic positive square wave at 0.1 Hz with a duty cycle of 10%, sent to the Porti7 through a dedicated isolated digital input, and to Philips iE33 through its AUX channel. The trigger has two different amplitudes for the two devices: 4 V for Porti7 and 100 mV for iE33. The trigger was recorded on the Porti7 as a digital signal, synchronised with the other inputs, whereas it was sampled as an analogue signal by the iE33, producing a green trace on the lowest part of the screen, normally reserved to the electrophysiological input when the ECG leads of the ultrasound device are connected to a patient. The synchronisation between the trigger signal and PWD trace, as shown in the iE33 video, is guaranteed by the manufacturer because the AUX signal is conceived to provide a reference electrophysiological signal to the cardiologist during an echocardiography (e.g. as a gating signal).

The synchronisation of PWD and electrophysiological recordings acquired with the Porti7 system was accomplished in post-processing using a custom MATLAB interface. The custom interface presented to the user the PWD video frames with the embedded trigger signal. The user could select how to synchronise the recordings on the first rising or falling edge of the trigger. The user could then scroll the video frames to identify the exact frame where the trigger started to rise or fall, then mark the frame number. Finally, the PWD video and electrophysiological and respiration signals were cut to exactly represent the same epochs of the signals in the two modalities.

This offline synchronisation method was studied with the technicians of the iE33 device and was determined to represent the only accurate way to synchronise the two devices because the other approaches work for only small examination snapshots or frames. The method accuracy was thoroughly tested on different signals prior starting the data acquisition, to ensure the correctness of the results.

## Data Records

The dataset is freely available on PhysioNet with the name Non-Invasive Multimodal Foetal ECG-Doppler Dataset for Antenatal Cardiology Research (NInFEA)^35^. The dataset includes 60 entries from 39 pregnant women volunteers between the 21st and 27th week of gestation. The signal length varies from 7.50 s to 119.80 s (average 30.6 s $\pm$ 20.6 s). The length of each trace is listed in Table 2. The clinical information about each data record included in the dataset are listed in Table 3. Two files are available for every data record: the PWD trace as an image in the standard Bitmap (.bmp) format and signals from the Porti7 device. The latter is stored in a custom binary format (.bin) described in Table 4, to ensure maximum compatibility. For PhysioNet compatibility, the standard WFDB software package format, which can be read and converted to other formats using functions such as rdsamp from this package^62^, is also provided in the online dataset.

**Table 2:** Duration of the signal segments that compose the dataset.

Signals from same participant	# signal segments	first trace duration [s]	second trace duration [s]	third trace duration [s]
1−3	3	28.07	28.64	53.06
4−5	2	44.33	47.31	
6−7	2	43.43	52.60	
8	1	13.02		
9	1	12.58		
10−11	2	37.24	17.43	
12	1	7.50		
13−14	2	23.92	45.60	
15−16	2	58.74	14.73	
17	1	31.11		
18	1	25.88		
19	1	28.87		
20−21	2	46.90	46.44	
22	1	14.32		
23	1	29.31		
24	1	64.37		
25	1	119.78		
26	1	76.04		
27	1	41.69		
28−29	2	15.39	13.22	
30	1	57.21		
31−33	3	13.88	20.88	28.10
34	1	15.46		
35−36	2	12.68	11.88	
37−38	2	25.43	10.10	
39	1	15.86		
40−41	2	12.51	25.09	
42	1	10.97		
43−44	2	50.15	34.66	
45	1	22.15		
46	1	54.80		
47−48	2	11.78	15.39	
49−50	2	9.07	46.74	
51−52	2	24.32	12.90	
53−54	2	11.44	12.25	
55−56	2	13.75	42.46	
57	1	12.88		
58	1	59.57		
59−60	2	23.99	32.93

**Table 3:** Clinical information about mothers and foetuses.

Signals	Gestational week	Mother’s age [y]	Mother’s height [m]	Mother’s weight [kg]	Prev. pregnancies	High-Risk Pregnancy	Gestational diabetes	Pre-eclampsia	Risk Factors	Foetal Presentation
1−3	27	27	1.7	60	0	no	no	no		vertex, OT
4−5	25	34	1.6	60	1	no	no	no	${}^{1}$	vertex, ROT
6−7	21 + 1	30	1.5	53	0	yes	no	no	${}^{2}$	vertex, ROT
8	22 + 4	32	1.65	57	0	yes	no	no	${}^{6}$	vertex, LOT
9	24	38	1.7	66	1	no	no	no		vertex, LOT
10−11	24 + 4	35	1.6	68	2	no	no	no		breech, LST
12	25 + 4	38	1.5	71	1	yes	yes ${}^{3}$	no		breech, LSA
13−14	21 + 5	29	1.6	64	0	no	no	no		breech, RSP
15−16	25 + 2	34	1.58	67	1	no	no	no		vertex, ROP
17	24	41	1.55	63	2	no	no	no		vertex, ROP
18	26 + 6	37	1.7	83	2	yes	no	no		breech, RST
19	22 + 4	36	1.6	73	3	yes	no	no ${}^{5}$		breech, LSP
20−21	26 + 2	30	1.51	71.5	0	no	no	no		breech, LST
22	25 + 1	28	1.51	61.8	0	no	no	no		vertex, LOP
23	24 + 1	33	1.6	69	0	no	no	no		breech, RSP
24	26 + 6	32	1.6	78	0	yes	no ${}^{4}$	no		breech, LSP
25	26 + 3	36	1.75	69	0	no	no	no		vertex, LOP
26	24 + 1	38	1.6	63	1	no	no	no		vertex, LOP
27	27 + 1	42	1.48	45	0	yes	yes ${}^{3}$	no		vertex, LOP
28−29	26 + 6	39	1.6	69	0	no	no	no		vertex, ROP
30	22 + 3	31	1.6	60	1	no	no	no		breech, LSP
31−33	27 + 5	31	1,56	65	0	no	no	no		vertex, ROP
34	25	39	1,6	63	3 ${}^{\dagger }$	yes	yes	no		breech, RSP
35−36	27	37	1,63	64	1	no	no	no		vertex, ROP
37−38	25 + 1	24	1,73	85	1 ${}^{\dagger }$	yes	no	no		vertex, LOP
39	24	20	1,68	91	0	no	no	no		vertex, OA
40−41	21 + 3	43	1,61	62	yes	no	no	no		breech, RSP
42	24 + 6	31	1,5	60	0	no	no	no		breech, LSP
43−44	23 + 4	29	1,57	63	0	no	no	no		vertex, ROP
45	23	37	1,53	60	2 + 1 ${}^{\dagger }$	no	no	no		vertex, OP
46	24 + 4	39	1,64	55	1	no	no	no		breech, LSP
47−48	21 + 1	34	1,59	47	1	yes	no	no		vertex, LP
49−50	22	29	1,65	77	1	yes	no	no		breech, LSP
51−52	24 + 2	36	1,53	49	1	no	no	no		vertex, LOP
53−54	21	40	1,55	54	1 ${}^{\dagger }$	yes	no	no		vertex, LOP
55−56	25	25	1,65	60	0	no	no	no		breech, LSP
57	23 + 6	30	1,55	70	0	no	no	no		vertex, OA
58	23	26	1,7	52	2 ${}^{\dagger }$	yes	no	no		vertex, ROP
59−60	27 + 3	31	1,68	63	1	no	no	no		vertex, LOP

Regarding the foetus, the presentation is first listed followed by the its position, in particular: L: left, R: right, O: occiput, S: sacrum, T: transverse, P: posterior A: anterior. ${}^{\dagger }$:number of previous abortions.

^1^daughter with pulmonary hypertension related to surfactant deficiency disorder; ^2^mother affected by congenital heart disease; ^3^insulin-treated; ^4^hyper-insulinemic; ^5^gestational hypertension; ^6^fibrosis.

**Table 4:** The custom binary format used for storing the data files.

Description	Number of bytes	Precision*
Sampling frequency (*f*_*s*_)	8	64-bit double precision floating point (double)
Number of channels (*r*)	8	64-bit unsigned integer (uint64)
Number of time samples (*c*)	8	64-bit unsigned integer (uint64)
Data**	8 × *r* × *c*	64-bit double precision floating point (double)

*The machine format of all entries is IEEE Little Endian.

**The 8-byte data words of all channels are written sample-wise starting from channel 1 to channel *c*

The raw electrophysiological and respiration signals are in the n.bin files (*n* = 1, …, 60), and the WFDB compatible signals are in the n.dat files accompanied by their corresponding n.hea header files. In order to guarantee the maximum precision in the representation of the signals in 32-bit WFDB binary format, the DC offset was removed in this format and the DC value for each channel was saved in the.hea files, for possible use.

The first 27 rows are the channels presented in Fig. 4, row 32 is the maternal respiration signal, row 33 is an internal saw-tooth signal and row 34 is associated with the trigger signal used for synchronisation. The signal processing for data use is completely up to the user because of the raw signals. This gives the final user the possibility to obtain maximum control over the processing applied to the data, including the possibility to digitally combine different unipolar channels to produce differential channels with diverse orientation in space. Given the Porti7 device is dc-coupled, the user is expected to implement baseline wandering removal first because this artefact can be intense depending on maternal respiration and small movements, beyond the pressure effect of the ultrasound probe on the maternal abdomen.

As part of a continuous research project, the dataset will be expanded with more recordings using the same homogeneous setup.

## Technical Validation

We demonstrate some important parameters that could help a researcher in the selection and use of our data for the development and assessment of novel algorithms for fECG extraction and processing, PWD analysis and foetal cardiac physiology studies to assess the quality of the presented dataset. As a result, the fECG signal was extracted from the raw data exploiting open-source algorithms and the procedure is described in the following paragraph.

### Foetal ECG extraction

Various methods have been developed for non-invasive fECG extraction^26^. Among the existing methods, multi-channel techniques based on variants of *blind and semi-blind source separation* methods are the most robust because they can can be used in different gestation ages, different electrode configurations and foetal positions. Herein, we used the most recent developments in this field that apply to multi-channel recordings in low-rank and low SNR, and are robust to maternal and/or fECG morphological variations because of anomalies and temporal variations (e.g. because of foetal motion and rotation during the acquisition session)^63–65^.

**Fig. 8:**
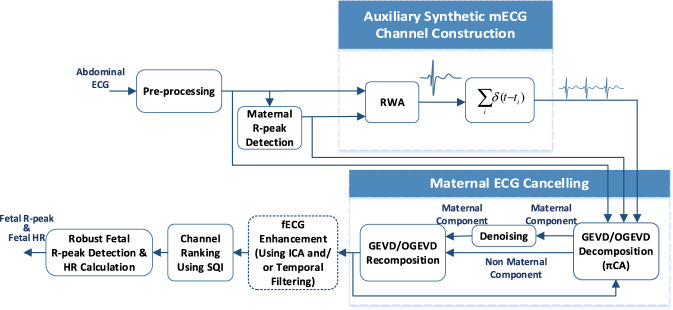
The overall foetal ECG and heart rate extraction scheme, adopted from^64^.

The overall steps of the algorithm are depicted in Fig. 8. Accordingly, the raw multi-channel signals are first pre-processed by a band-pass filter with a pass-band between 0.05 Hz and 250 Hz. The maternal R-peaks are then detected from one of the reference thoracic channels. These peaks are used in a so-called deflation algorithm based on *periodic component analysis* to remove maternal ECG components^66^. The resultant multi-channel signals are given to a blind-source separation algorithm, known as *joint approximate diagonalization of eigenmatrices* (JADE), using the jadeR.m MATLAB implementation by J. F. Cardoso^67^ for extracting the fECG components. Since JADE does not guarantee the order of the fECG in the extracted channels, an automatic channel ranking algorithm developed in^64^ is used for the automatic ranking of the foetal components. The automatic ranking results were confirmed by an expert, for every participant.

The R-peaks of the foetus were next detected from the fECG channel, using a matched filter with predefined templates^64^. The resulting foetal R-peaks were eventually used for extracting the average fECG morphology. The sample codes and functions for performing the noted fECG extraction scheme are online available in the *open-source electrophysiological toolbox* (OSET)^34^.

**Fig. 9:**
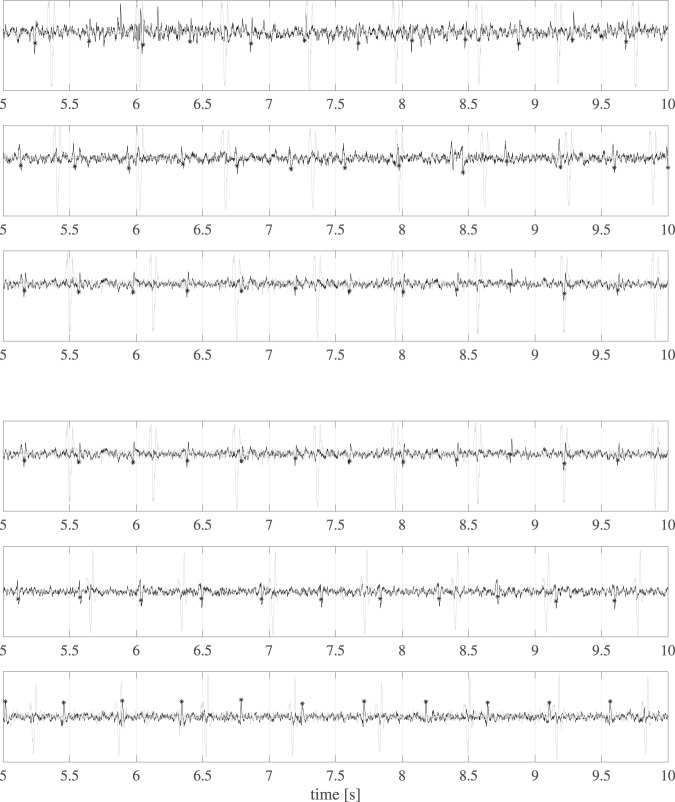
The extracted foetal ECG traces from the first six recordings between the 5^*th*^ to the 10^*th*^ second of recording. In the figure, the foetal ECG is black whereas the reference maternal ECG is light grey. The black asterisks highlight the foetal peaks detected by the exploited algorithm. A blind-source-based separation process was used so the foetal ECG amplitude did not have a physical meaning. The amplitude of the reference thoracic maternal ECG was normalized and rescaled for display purposes only.

Some examples of foetal ECG traces extracted with this method from the first six recordings are shown in Fig. 9.

### Dataset quality assessment

**Fig. 10:**
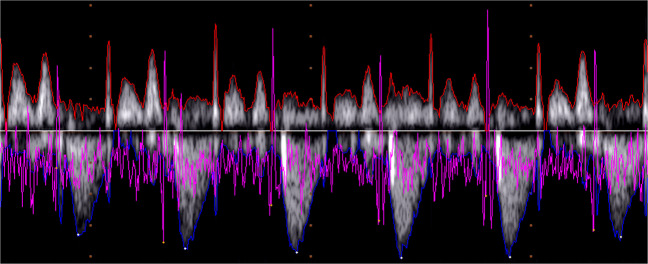
Example of synchronised foetal ECG (pink), the PWD signal (grey), and lower (blue) and upper (red) PWD envelopes (the first six beats from the 4^*th*^ trace).

The capability to extract the foetal QRS complexes by exploiting the PWD as a reference signal to check the actual occurrence of a ventricular activation in the foetal heart was analysed to provide some quantitative data to support the quality of the dataset. Even though atrial contraction could occur even without a physiological P wave, the same is not true for the ventricular contraction in relation with the QRS. This is the only option to check the actual effectiveness of a fECG extraction algorithm in early pregnancy, which is not available in any of the available datasets for non-invasive fECG analysis and processing. Nevertheless, considering that there is no tight timing relationship between the mechanical activation of the foetal heart and the originating depolarisation signal, it was impossible to identify a rigorous criterion for the acceptability of a possible foetal QRS occurrence that is different from the visual assessment by the experts. For a visual comparison between the different modalities, we can consider Fig. 10, where a physiological pattern (from the 4^*th*^ trace and showing the first six beats) is shown (the fECG was extracted with the previously described procedure).

Foetal V-peaks in the PWD were then labelled by visual inspection whereas, on the fECG signal, the R-peaks were automatically obtained by the OSET toolbox, after signal extraction with the aforementioned method^34^. The foetal QRS detection performance was estimated versus the V-peak occurrence by considering clinically reasonable distances between them (less then 200 ms). The quantitative parameters considered for this assessment were: Accuracy (Acc), Sensitivity (Se), and Positive predictive value (PPV), computed as:

3 $${\rm{Acc}}=\frac{{\rm{TP}}}{{\rm{TP}}+{\rm{FP}}+{\rm{FN}}}$$

4 $${\rm{Se}}=\frac{{\rm{TP}}}{{\rm{TP}}+{\rm{FN}}}$$

5 $${\rm{PPV}}=\frac{{\rm{TP}}}{{\rm{TP}}+{\rm{FP}}}$$

where TP is the number of true positive detections, FP is the number of false positive detections, and FN is the number of false negative detections. Note that since the detected fECG R-peaks are compared with the V-peaks in the PWD, *accuracy* does not account for the *true negatives* and is identical with the *critical success index* in the context of machine learning, which accounts for the total hits divided by the number of hits plus false alarms and misses.

The fECG and acceptable QRS detection were extractable from 95.5% of the data segments (the percentage of the data segments without considerable noise contamination), using the semi-supervised procedure described above. Table 5 lists the evaluation results of the R-peak detection. The median Acc, Se and PPV values were 0.79, 0.97 and 0.81, respectively.

**Table 5:** Evaluation results of the automatic foetal QRS detection.

Sig. #	Acc	Se	PPV	Sig. #	Acc	Se	PPV
**1**	0.90	0.97	0.93	**31**	0.66	0.91	0.70
**2**	0.96	0.97	0.99	**32**	0.85	0.98	0.87
**3**	0.72	0.99	0.73	**33**	0.46	1.00	0.46
**4**	0.86	0.99	0.87	**34**	0.71	0.97	0.73
**5**	0.84	0.98	0.86	**35**	0.74	0.96	0.77
**6**	0.93	0.99	0.94	**36**	0.97	0.97	1.00
**7**	0.23	0.93	0.23	**37**	0.37	0.92	0.38
**8**	0.75	0.93	0.79	**38**	0.93	0.96	0.96
**9**	0.82	0.94	0.86	**39**	0.68	0.93	0.72
**10**	0.43	0.97	0.44	**40**	0.97	0.97	1.00
**11**	0.33	0.88	0.35	**41**	0.38	0.92	0.40
**12**	0.95	0.95	1.00	**42**	0.88	0.92	0.96
**13**	0.90	0.97	0.93	**43**	0.64	0.99	0.65
**14**	0.88	0.99	0.89	**44**	0.54	0.96	0.55
**15**	0.23	0.97	0.23	**45**	0.84	0.98	0.86
**16**	0.82	0.97	0.85	**46**	0.21	0.93	0.21
**17**	0.75	0.97	0.77	**47**	0.68	0.95	0.70
**18**	0.88	0.98	0.90	**48**	0.53	0.95	0.54
**19**	0.97	0.97	1.00	**49**	0.19	0.80	0.20
**20**	0.95	0.99	0.96	**50**	0.50	0.96	0.51
**21**	0.26	0.97	0.26	**51**	0.46	0.97	0.47
**22**	0.89	0.94	0.94	**52**	0.80	0.93	0.85
**23**	0.80	0.98	0.81	**53**	0.86	0.96	0.89
**24**	0.87	0.98	0.88	**54**	0.81	0.93	0.86
**25**	0.63	0.99	0.64	**55**	0.84	0.96	0.87
**26**	0.51	0.99	0.52	**56**	0.71	0.97	0.73
**27**	0.33	0.97	0.33	**57**	0.82	0.97	0.85
**28**	0.78	0.94	0.83	**58**	0.97	0.99	0.98
**29**	0.79	0.96	0.81	**59**	0.63	0.97	0.64
**30**	0.20	0.97	0.20	**60**	0.89	0.99	0.90

Then, instantaneous fHR from the fECG and PWD were evaluated and compared. The comparison was mainly qualitative and affected by both the different time resolutions of the two signals and limited accuracy in identifying fiducial points on the PWD signals. The average value for each trace was derived from the instantaneous values.

**Fig. 11:**
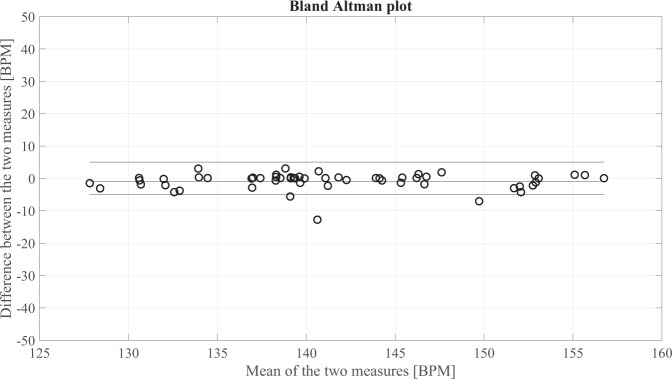
Bland-Altman plot for the foetal heart rate obtained from non-invasive fECG and from PWD traces.

**Fig. 12:**
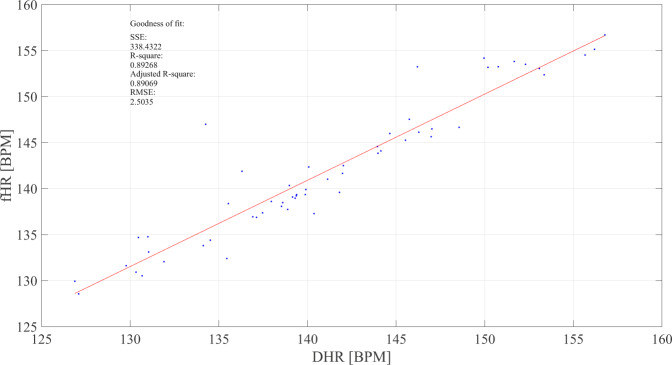
Correlation between mean foetal heart rate achieved on each trace from fECG (fHR) and PWD (DHR).

**Fig. 13:**
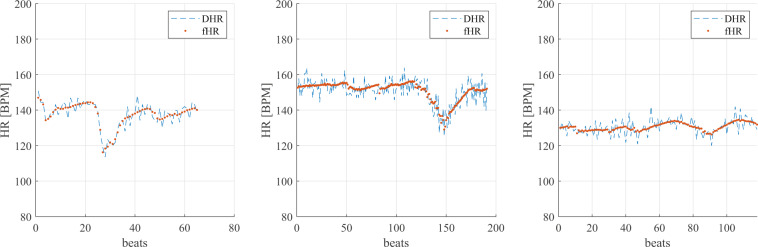
Examples of foetal heart rate obtained from the foetal ECG (fHR) and PWD trace (DHR) for three participants.

Agreement between the simultaneous measurements of fHR was assessed by computing the correlation coefficient and related p-value. Bland-Altman plots were also constructed^68^. The Bland-Altman plot, depicted in Fig. 11, and the correlation analysis in Fig. 12, confirm the agreement between the mean HR calculated on the two different modalities, PWD and fECG. The correlation coefficient *r*^2^ = 0.89 indicates a high fit. The explanation of the differences can be understood from Fig. 13, which shows three different tacograms (HR time series), belonging to three different foetuses, along with the correlation study results between the points of the two traces. The two methods always share the same range and trend of HRs. In Fig. 13, the leftmost plot reveals an *r*^2^ = 0.80 showing a high correlation; however, in the rightmost plot, the correlation coefficient dropped to *r*^2^ = 0.50, even though the two HR signals follow the same trend. This change is mainly attributed to the different sampling frequency of the two signals because of the steps used for PWD envelope extraction, resulting in a high variability (‘micro-fluctuations’) in the HR and poor precision in the identification of successive V-peaks in the PWD.

## Discussion

This dataset is particularly important for scientists working on foetal ECG extraction from non-invasive recordings because it is the first multimodal dataset in early pregnancy, including non-invasive electrophysiological, maternal respiration and Doppler signals. In particular, the latter is able to provide a non-invasive trustable reference for foetal heart activity in early pregnancy when a scalp electrode cannot be adopted. As no public datasets with synchronised non-invasive electropysiological and echocardiographic signals from the foetal heart are available, this dataset could help develop more insight into clinical knowledge about foetal cardiac function, which is still far from that available for adult cardiology^69^. As a result, the expected users of this dataset are biomedical engineers working on signal processing as well as scientists interested to the study of foetal cardiology.

In the selection of the reference signal, the choice of foetal echocardiography, and in particular the 2D PWD modality, has significant reflections on some aspects of the dataset. Even though there is no strict timing relationship between the electrical and mechanical activity of the heart, neither in an adult nor foetus, echocardiography can provide hints for guessing the electrophysiological activity during routine evaluation of the fHR and rhythm^5^. Echocardiography is normally used for the detailed analysis of the foetal heart morphology and function^48^. PWD and M-mode modalities represent the routine evaluation of fHR and rhythm^5^ however, the PWD technique is preferred when the main objective is to measure the atrial and ventricular mechanical activity in terms of time intervals. The PWD in this dataset represents the ground truth, so that accuracy in the time measurement plays a major role.

Even after the advances in 2D modalities, Doppler echocardiography is still a fundamental part of the cardiovascular echocardiographic examination^70^. In particular, PWD is suggested for the assessment of cardiac arrhythmia^44^ and valvular/blood-flow dysfunctions^8^. Several studies used PWD alone^45^ or with the M-mode^42^ as a reference and complement for the foetal ECG or magnetocardiography. Some synchronised comparisons have been reported in the literature for the latter^45^; however, no direct comparison between the two modalities has been reported, apart from the HR with 1D Doppler^71,72^. Given the complex and changing nature of the 1D Doppler signal^73^, 1D Doppler ultrasound is not adopted in clinical routines, except for cardiotocography^47^, which is mainly used in late pregnancy for continuous HR monitoring. However, this technique cannot provide a signal useful for the detailed analysis of atrial and ventricular activity, i.e. the ultrasound signal is used only to compute the HR, which usually exploits processing techniques that introduce an averaging effect that in turn could reduce the fHR accuracy and beat-to-beat variability^74^. For this reason, it was impossible to adopt cardiotocography as a reference signal in our dataset.

Although these results justify the choice of PWD as reference signal for the foetal heart activity in early pregnancy, there is a relevant reverberation on the dataset: the limited duration of the traces requires a meaningful reference signal to reveal details about atrial and ventricular activity. By using PWD (or M-mode)^42,45^ it is impossible to obtain minutes long recordings without losing the sample volume and then any clinical relevance of the recorded ultrasound signal. The main reason is that the foetus in utero has high mobility in early pregnancy compared with late pregnancy when the size of the foetal heart is significantly larger (at 21 weeks, the inner diameter of the left ventricle is less than 1 cm^75^). In such conditions, even small foetal movements do not provide a well-shaped morphology of PWD (or M-mode) without probe repositioning. For this reason, for some participants we provided up to three segments extracted from the same continuous recording, after probe repositioning. For the same reason, two out of 60 signals have a duration less than 10 seconds. These short traces were included because they can be valuable for some algorithms not requiring long initial training periods. The longest signal is approximately two minutes, which is a unique case. With such duration, the signals in the dataset are not usable for the study of fHR variability. In such cases, non-invasive fECG or cardiotocography (in late pregnancy) is better.

A further consideration is the number of channels and the positioning of the sensors. The dataset implements a large number of unipolar abdominal channels, providing the user the possibility to reproduce several state-of-the-art positionings conceived by other researchers. This is significantly important for studies on fECG extraction from non-invasive recordings because the impact of this variable on the fECG amplitude at the electrodes has been demonstrated^76^. By using the proposed dataset, the interested researchers can test different configurations on the same signals, enabling comparison over more than 60 signals. Moreover, studies on the inverse problem, best positioning, foetal heart localisation and value of geometric transforms in the lead system reconstruction can be performed on the available data. This dataset, which is the outcome of more than ten years of research finalised to the identification of the best acquisition setup (device, electrodes positioning, electrodes, multimodal measurement and synchronisation, etc) was expressly developed to help other researchers perform their studies without the the cost, technical and time burden associated with data collection. This dataset will hopefully foster research to provide new methods aimed at addressing the open research issue of optimal foetal ECG extraction.

## Usage Notes

The hereby presented dataset and processing tools are provided for public use and may be used with proper citation to the current paper.

## Data Availability

Beyond the data, a small library of MATLAB (The Mathworks, MA, USA) custom functions accompanies the dataset. In particular, a binary file reader for MATLAB is provided, enabling loading the signals acquired with the Porti7 electrophysiological recording system in a MATLAB variable. Using the source code, analogous readers in different programming languages can be also developed. We also provided a graphical user interface enabling simultaneous scrolling of the long PWD image (first loaded as.bmp file) and all the related Porti7 channels and internal signal (in.bin format). This interface can be used to browse the raw data easily. A scientist can modify its source code to show the fECG signals extracted by the preferred method without any limitation. This feature was not added in this version to avoid any bias in the data evaluation. Additionally, the envelope extraction function described in this paper is available for PWD processing. In this work, we presented the dataset and discussed its potentialities for scientific and technological advancements in the field. As proof of concept, we provided figures of merit enabling researchers to quantitatively evaluate the dataset. The code for fECG extraction and processing is available in the *open-source electrophysiological toolbox* (OSET)^34^.
